# Transforming Households with Refraction and Innovative Financial Technology (THRIFT): study protocol for a randomised controlled trial of vision interventions and online banking among the elderly in Kurigram

**DOI:** 10.1136/bmjopen-2024-085083

**Published:** 2024-12-23

**Authors:** Sharmin Akter Shitol, Ishrat Binte Aftab, Prabhath Piyasena, Lynne Lohfeld, Sridevi Rayasam, Nagamani Challa, Payal Sangani, Lovemore Nyasha Sigwadhi, H M Masudur Rahman, Rohit C Khanna, Ving Fai Chan, Mrittika Barua, Sonia Pant, Achyuta Adhvaryu, Anant Nyshadham, Malabika Sarker, Asha Latha Mettla, Enam Haque, Graeme MacKenzie, Sadiq Alam, Ella Gudwin, Mike Clarke, Abu Shonchoy, Atonu Rabbani, Nathan Congdon

**Affiliations:** 1 BRAC University James P Grant School of Public Health, Dhaka, Bangladesh; 2 School of Medicine, Dentistry and Biomedical Sciences, Centre for Public Health, Queen's University Belfast, Belfast, UK; 3 School of Medicine, Dentistry and Biomedical Sciences, Centre for Public Health, Queen's University Belfast School of Medicine Dentistry and Biomedical Sciences, Belfast, UK; 4 Centre for Public Health, Queen's University Belfast School of Medicine Dentistry and Biomedical Sciences, Belfast, UK; 5 Clinical Trials Unit, LV Prasad Eye Institute, Hyderabad, Telangana, India; 6 Department of Global Health, Stellenbosch University Faculty of Medicine and Health Sciences, Cape Town, South Africa; 7 MOMODa Foundation, Dhaka, Bangladesh; 8 Allen Foster Community Eye Health Research Centre, Gullapalli Pratibha Rao International Centre for Advancement of Rural Eye care, L V Prasad Eye Institute, Hyderabad, Telangana, India; 9 Brien Holden Eye Research Centre, L.V. Prasad Eye Institute, Hyderabad, Telangana, India; 10 University of New South Wales School of Optometry and Vision Science, Sydney, New South Wales, Australia; 11 University of Rochester School of Medicine and Dentistry, Rochester, New York, USA; 12 Centre for Public Health, Queen's University Belfast, Belfast, UK; 13 VisionSpring, New York, New York, USA; 14 University of California San Diego, La Jolla, California, USA; 15 University of Michigan, Ann Arbor, Michigan, USA; 16 Heidelberg University, Heidelberg, Germany; 17 Allen Foster Community Eye Health Research Centre, ICARE, L V Prasad Eye Institute, Hyderabad, Andhra Pradesh, India; 18 Riemann Limited, London, UK; 19 Metakave, Dhaka, Bangladesh; 20 Florida International University, Miami, Florida, USA; 21 Department of Economics, University of Dhaka, Dhaka, Dhaka District, Bangladesh; 22 School of Medicine, Dentistry and Biomedical Sciences, Centre for Public Health, Queen's University Belfast MDBS, Belfast, UK; 23 Preventive Ophthalmology, Zhongshan Ophthalmic Centre, Sun Yat-Sen University, Guangdong, Guangdong, China; 24 ORBIS International, New York, New York, USA

**Keywords:** Internet, OPHTHALMOLOGY, EPIDEMIOLOGIC STUDIES

## Abstract

**ABSTRACT:**

**Introduction:**

Presbyopia, difficulty in seeing close-ups, affects a billion people globally. Mobile financial services (MFS) have been mandated since January 2021 for Bangladesh government social safety net payments, including old age allowance (OAA) and widow allowance (WA). We report the protocol for the Transforming Households with Refraction and Innovative Financial Technology randomised trial assessing the impact on the use of online banking of providing presbyopic safety net beneficiaries with reading glasses, and brief smartphone and mobile banking app training.

**Methods and analyses:**

Eligible participants (n=484) are OAA (men aged 65–70 years; women aged 62–70) or WA recipients (women aged 48–60) with presbyopia as their only vision problem, passing a smartphone-based test of numeracy, cognition and dexterity, and not currently owning a smartphone or independently using MFS. All participants receive smartphones loaded with a mobile banking app and a transaction-tracking app and are randomised 1:1 to receive immediate free near-vision glasses and half-day training for smartphone and banking app use (intervention), or glasses and training 12 months later (control). The primary outcome is the mean quarterly number of mobile bank transactions over the 12-month follow-up period, comparing study groups, with and without adjustment. Secondary outcomes include food security, healthcare access and social connectedness.

**Ethics and dissemination:**

The protocol was approved by ethics committees at Queen’s University Belfast (reference #MHLS22_69) and BRAC James P Grant School of Public Health (reference #IRB-21 August’22-028). The trial is conducted in accordance with the Declaration of Helsinki and national regulations in Bangladesh, and results will be published in open-access, peer-reviewed journals.

**Trial registration number:**

NCT05510687; ClinicalTrials.gov.

**STRENGTHS AND LIMITATIONS OF THIS STUDY**

- Transforming Households with Refraction and Innovative Financial Technology is the first randomised controlled trial to evaluate the impact of providing near-vision glasses on presbyopic old age allowance and widow allowance beneficiaries’ use of mobile banking.
- A favourable trial outcome will support the integration of near-vision services into similar mobile financial services programmes in other regions, aiming to enhance their effectiveness in promoting financial inclusion and reducing poverty.
- This study focuses on online banking for the elderly, a topic of strong interest to governments and multilaterals such as the World Bank.
- Other people in the households of the beneficiaries may use the study mobile phone, potentially affecting the results.
- Not all beneficiaries of social safety net payments have the cognitive ability, numeracy or dexterity for smartphone use, potentially limiting the applicability of results.

## Introduction

 The most common cause of visual impairment worldwide is uncorrected presbyopia,^1^ the inability to focus on nearby objects unaided with 90% of the burden falling in low and middle income countries (LMICs).^2^ Recent estimates indicate that presbyopia affects more than one billion people worldwide, beginning during the most productive working years of 35 and above.^3^ Studies in LMICs have found that uncorrected presbyopia reduces quality of life, and most commonly affects women, those with lower education levels and the elderly, and encourages dependence on others for near-vision activities.^4^,^6^ Conversely, treatment of presbyopia is trial-proven to increase workplace productivity, with effect sizes larger than for other health interventions.^7^

There is a substantial burden of uncorrected presbyopia in Bangladesh,^8^ in part due to a rapidly ageing population. The 2022 census found that 9.3% of the population (15.3 million people) were 60 years old or above,^9^ and this proportion is estimated to reach 22% by 2050.^10^ Virtually all of these persons would be expected to be presbyopic as part of normal ageing.

To help meet the needs of vulnerable people in Bangladesh, the government provides regular cash transfers for poor elderly men (>65 years) and women (>62 years) through the old age allowance (OAA) and women who are widowed, deserted or destitute (>18 years) through the widow allowance (WA) programme. These programmes are administered by the Department of Social Services (DSS) under the Ministry of Social Welfare and deliver monthly payments of 500 BDT (about US$4.63). Till recently, beneficiaries had to travel each quarter to designated bank branches and wait in long queues to draw the payments personally.^11^

Recently, mobile financial services (MFS) have become popular in Bangladesh, as in other LMICs, as a means of improving access to formal banking services and increasing financial inclusion.^12^ In 2021, the government of Bangladesh began disbursing OAA and WA payments directly through MFS platforms offered by two leading local providers: Nagad and bKash.^13^ The aim of this policy was to reduce transaction and time costs, and avoid the involvement of a third party for payment collection.^11^

Older adults in LMICs face a variety of challenges when using mobile phones, including uncorrected near-vision impairment,^5 14^ lack of understanding of the phone itself and associated apps, illiteracy or innumeracy,^15^ insufficient manual dexterity^16^ and concerns over unauthorised use by others.^17 18^ These issues can hinder the uptake of MFS for managing their finances.

The Transforming Households with Refraction and Innovative Financial Technology (THRIFT) trial will evaluate the impact of providing near-vision glasses on presbyopic OAA and WA beneficiaries’ use of mobile banking. The current report follows the SPIRIT guidelines (https://www.spirit-statement.org/) and presents the THRIFT trial protocol (https://clinicaltrials.gov/study/NCT05510687?locStr=Bangladesh&country=Bangladesh&intr=presbyopia&rank=1), methodology, proposed outcome measures and study hypotheses.

THRIFT is the first trial to assess the effect of the provision of presbyopic glasses on the use of mobile banking among elderly persons in LMICs. This research is significant as the burden of uncorrected presbyopia is high^2^ and growing due to the ageing of the global population,^10^ while mobile banking is becoming increasingly important and widespread.

Access to digital financial services (DFS), such as bKash in Bangladesh and mPesa in Kenya, is widely considered an effective tool to combat financial exclusion and poverty.^19 20^ However, it is known that uncorrected presbyopia significantly hinders smartphone use.^14^ The World Bank and other multilateral organisations have expressed strong interest in replicating Bangladesh’s use of MFS platforms to distribute social safety net payments to the elderly. A positive trial result will provide evidence in favour of the incorporation of near-vision services into similar MFS programmes elsewhere in order to maximise their potential for financial inclusion and poverty alleviation.

## Materials and methods

THRIFT is a mixed-methods, investigator-masked randomised controlled trial (RCT). Qualitative formative research (interviews with members of stakeholder groups) will precede the trial to examine contextual and cultural factors that may affect the uptake of the intervention.

### Setting

The research will be conducted in Kurigram Sadar and Nageshwari, two subdistricts within the Kurigram district located in northern Bangladesh. Kurigram was chosen due to the presence of bKash, the MFS partner for government social safety net programmes in this region. The two subdistricts were chosen in collaboration with VisionSpring, the implementing partner, and their local collaborator, BRAC, which already conducts eyecare programmes in these areas. The targeted population in this economically disadvantaged region was anticipated to significantly benefit from improved access to social safety net payments.^21^ The start date for the study was 10 December 2023. Enrolment is now completed, and the study is in the follow-up phase, with an anticipated end date in June 2025.

### Participants

Participants in the trial are selected based on the following criteria.

#### Inclusion criteria

- Age verified by national identity card meeting the following criteria:
  - Male OAA recipients: 65–70 years old
  - Female OAA recipients: 62–70 years old
  - WA recipients: 48–60 years old
- Currently receiving digital OAA or WA payments from the government of Bangladesh
- Lived in Kurigram bKash catchment area for ≥3 years and plan to reside here for the study duration
- Presbyopia, defined as binocular presenting near vision of N6.3 or worse, correctable to N5 or better
- Total household assets in the bottom three quintiles of the screening cohort according to the study equity tool questionnaire
- Score ≥8 (out of 10) on a smartphone-based numeracy, dexterity and cognition screening test developed by the investigators
- Not currently using an MFS account independently

#### Exclusion criteria

- Internet connection speed <13 kbps measured by study personnel
- Near or distance vision impairment that cannot be resolved with presbyopic glasses

### Study recruitment

The research team received the list from the DSS identifying OAA and WA beneficiaries living in the Kurigram district. After meetings with local leaders, study teams affiliated with MOMODa Foundation (http://momodafoundation.org/about-us), a local non-government organisation, will conduct door-to-door visits to assess potential participants’ demographic characteristics, socioeconomic status, numeracy, manual dexterity and cognitive functioning required to perform basic financial functions on a mobile device. Respondents initially meeting eligibility criteria are offered an eye examination conducted by community health workers from the international non-governmental organisation VisionSpring (https://visionspring.org/about-us/our-story) to assess the presence of presbyopia meeting study criteria. Individuals meeting all the above inclusion criteria are offered the opportunity to consent for enrolment in THRIFT (online supplemental material 1).

### Randomisation, allocation and concealment

The randomisation sequence is generated by the study statistician at the Clinical Trials Unit (CTU) of the LV Prasad Eye Institute (LVPEI) in Hyderabad, India. Separate randomisation sequences are prepared in advance for each of the 12 possible strata, based on beneficiary group (OAA/WA), age (below or above the median within the beneficiary group), gender (male/female for OAA) and previous phone use (yes/no). Once the baseline data have been collected for all study participants, random allocation is carried out by the off-site statistician. The assignments at the individual level are then shared with the implementing partners. This will ensure allocation concealment as the treatment codes are not revealed till allocations are locked.^22^

### Study intervention

At the time of enrolment, all participants are provided with a smartphone preloaded with the bKash mobile banking application and a study-specific bank transaction tracking application (the ‘THRIFT App’). The latter monitors SMS records of incoming transactions on the participants’ phones. The data plans for each phone are remotely topped up on a monthly basis if intervention group participants state that they are complying with study spectacle use at follow-up visits occurring 1 and 6 months after glasses distribution. Glasses that have been damaged or lost are replaced at this time, and non-compliant participants will be told that their data plans and phone will only continue to be supplied if they wear the study spectacles.

All participants in THRIFT receive a complimentary eye examination and a pair of near glasses from VisionSpring, along with subsequent checks to ensure proper fit and optimal vision correction. Participants in the intervention group receive glasses at the beginning of the trial, whereas control participants will receive them at trial closeout. The power of the lenses needed to correct presbyopia is determined by the participant’s uncorrected near visual acuity, measured at a distance of 40 cm. Participants with binocular unaided near visual acuity of N6.3 (6/12), N10 (6/19), N12.5 (6/24), N20 (6/36) or N32 (6/60) require reading glasses with lens powers of 1.0D, 1.5D, 2.0D, 2.5D or 3.0D, respectively.

Providing placebo eyeglasses without power to control participants was not ethically acceptable in the view of the investigators and local stakeholders. This would lead study participants to erroneously conclude that glasses have no benefit and risk undermining the demand for vision services in the area.

Study personnel train participants allocated to the intervention group for 8 hours on using smartphones and the mobile banking app (bKash) at the beginning of the trial. Those allocated to the control group receive basic smartphone usage instruction at the beginning of the trial and the training on using smartphones and the mobile banking app at trial endline.

### Assessment of outcomes

The primary trial outcome is the mean quarterly number of mobile money transactions per participant using the MFS over the 12-month follow-up period. Data on such transactions are collected from two sources (table 1): data for outgoing transactions will be provided to the study team by bKash, while the study-specific THRIFT app records incoming mobile money transactions on participants’ phones. Study personnel responsible for collecting these data are masked to participants’ study group allocation.

**Table 1:** Primary and secondary outcomes with data type and measurement tools

	Data type	Section/survey tool	Collection period
Primary outcomes			
Mean quarterly number of mobile money transactions per participant	Continuous	THRIFT app, bKash app	12-month follow-up period
Mean quarterly number of interactions per participant	Continuous	bKash app	12-month follow-up period
Secondary outcomes			
Whether use of the application was facilitated by a bKash agent, family member or independently	Categorical	Baseline and endline surveys	Baseline and endline
Purchase of additional phones by study participant’s household	Categorical	Endline survey	Endline
Purchase of additional phones by study participant	Categorical	Endline survey	Endline
Intrahousehold resource sharing by the participant (as a percentage of total household consumption)	Continuous	Baseline and endline surveys^24^	Baseline and endline
Purchase of glasses	Categorical	Endline survey	Endline
Food security	Categorical	Food and Agriculture Organization’s food insecurity scale^25^	Baseline and endline
Role of study participant in household decision-making	Categorical	Baseline and endline surveys^23^	Baseline and endline
Subjective well-being of study participants	Continuous	Quality-of-Life Questionnaire (EuroQol 5-dimension 5-level)^26^ and Near Activity Visual Questionnaire^27 28^	Baseline and endline
Mobility of study participant	Continuous	International Physical Activity Questionnaire^29^	Baseline and endline
Social connectedness of study participant	Categorical	Baseline and endline surveys^30^	Baseline and endline
External remittances	Continuous	THRIFT app	Every quarter in the 12-month follow-up period
Self-reported incidence of theft or fraudulent use of money from the index participant’s account	Categorical	Baseline survey	12-month follow-up period

THRIFTTransforming Households with Refraction and Innovative Financial Technology

Secondary trial outcomes include whether the bKash app was used independently by the participant or with the help of a bKash agent, family member or other person (gathered through survey questions on mobile ownership and access); purchase of additional phones by participants or their household (survey questions on mobile ownership and access); degree of participant autonomy in household decision-making (survey questions on decision-making power in the household)^23^; intrahousehold resource sharing by the participant (as a percentage of total household consumption)^24^; food security (Food and Agriculture Organization’s food insecurity scale)^25^; subjective well-being of the participant (EuroQol 5-dimension 5-level (EQ-5D-5L) questionnaire for quality of life,^26^ Near Activity Visual Questionnaire (NAVQ) for presbyopia specific quality of life)^27 28^; mobility (International Physical Activity Questionnaire)^29^; social connectedness (Social Network Index)^30^ and self-reported incidence of theft or fraudulent use of the participant’s account (survey questions on money management and financial inclusion). With the exception of NAVQ, all of the above tools and questionnaires have been validated for use in Bangladesh (table 1).

### Data collection and management

**Figure 1:**
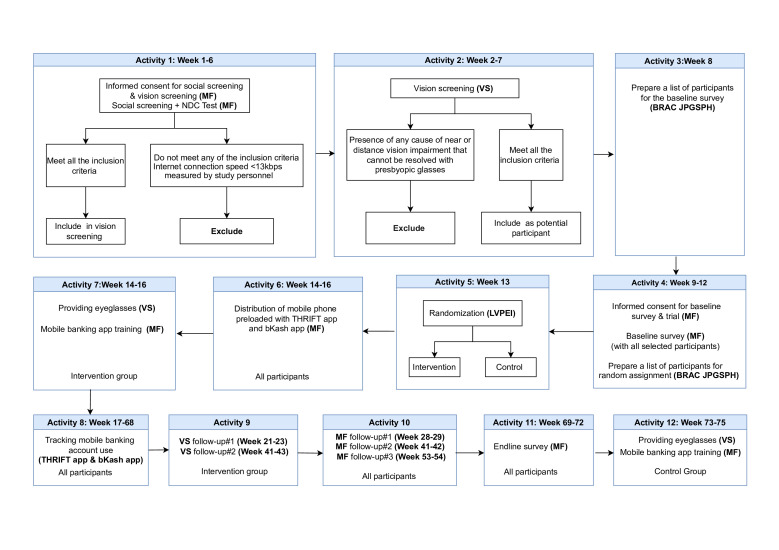
Flow chart showing the sequence of activities in Transforming Households with Refraction and Innovative Financial Technology. bKash app, mobile financial service application; BRAC JPGSPH, BRAC James P Grant School of Public Health; LVPEI, LV Prasad Eye Institute; MF, MOMODa Foundation; NDC, Neurology, dexterity, cognition; THRIFT app, Application developed by the trial team to track the mobile phone use of the participants; VS, VisionSpring.

Figure 1 displays the schedule of data collection and follow-up visits during the trial. Beneficiaries meeting the eligibility criteria are invited for the baseline and endline surveys including the primary and secondary outcome variables (table 1). MOMODa Foundation and VisionSpring conduct training sessions for all site staff involved in the trial, including those responsible for data collection and follow-up visits. The social screening survey and baseline survey data are captured electronically in the SurveyCTO database using a tablet. Data are reviewed and monitored regularly. Accuracy checks are carried out as follows: spot checks by the community health workers in the field, automated quality checks that flag outliers, suspicious distributions and means, and values that violate frequency or numeric thresholds, followed by a final check by the local research manager. Data from vision screening assessments are collected by trained community health workers and programme officers. Data are then entered into an Excel sheet by trained staff and evaluated for any entry errors before being shared with BRAC James P Grant School of Public Health (BRAC JPGSPH), the main academic partner in Bangladesh. Participants are identified on each form by their unique participant ID number. The final data will be shared with the CTU at LVPEI in Hyderabad, India, in Stata format for analysis. Analytical data will be stored at the LVPEI CTU for 10 years as stipulated by the funder Wellcome Trust.

Access to the data and the server used for data storage is restricted to authorised research personnel at BRAC JPGSPH, following the Research Governance Policies of LVPEI CTU (on behalf of the sponsor Queen’s University Belfast (QUB)), as well as the standard operating procedures implemented by BRAC JPGSPH for the management of physical and electronic research data. A comprehensive audit trail is maintained, allowing for the tracking of any changes made to the data, including the nature of the changes, dates of modification and the responsible personnel involved.

### Sample size

With a dropout rate of 15%, a total of 484 participants allocated 1:1 to the intervention and control groups is sufficient to detect a 15% increase (SD 0.32) in the mean number of quarterly transactions with the mobile banking app, with 90% power. Data for the number of transactions in the control group and the expected increase in transactions are based on our prior unpublished work on the impact of phone ownership on account transactions among OAA beneficiaries in Bangladesh.

### Statistical analysis

Baseline demographic and clinical characteristics will be summarised as means with SD in case of continuous normally distributed variables and medians with IQRs for non-normal distributed variables. Categorical variables will be summarised as numbers and frequencies (%). Testing for normality of data distributions will be based on the Shapiro-Wilk test.

An intention-to-treat approach will be applied in all analyses. The quarterly mean number of MFS transactions per beneficiary will be our primary outcome and will be compared between the intervention and control groups with and without adjustment for potential covariates using linear regression models. SEs will be clustered at the individual level for this analysis.

Study groups will be compared with and without adjustment for potential covariates using linear regression for all continuous secondary outcomes and mean differences and SEs will be reported. All continuous outcomes will be checked for normality and appropriate transformations used as needed. For binary outcomes, a binomial regression with a log-link will be used to estimate the relative risk and its CI; and a binomial model with identity link used to estimate the risk difference and its CI. In the case of non-convergence of the binomial model with a log-link, a Poisson model with robust SEs will be fitted. If the binomial model with the identity link does not converge, then only a relative risk will be reported. If neither the log nor the identity link converge, we will use the logistic link and report ORs.

A p value (two-tailed) <0.05 will be considered statistically significant. Stata (Stata Corp, College Station, Texas, USA) will be used for statistical analyses.

### Economic evaluation

We will conduct an economic evaluation of the intervention using cost-effectiveness analysis from a programmatic perspective. Specifically, our aim is to calculate the incremental cost per quality-adjusted life-year (QALY) gained based on EQ-5D-5L responses.^31 32^ The EQ-5D-5L is a widely used measure of QALY, recommended by the National Institute for Health and Care Excellence for use in the UK. Responses to EQ-5D-5L will be converted to utility scores using appropriate weights. This data will be collected at baseline before randomisation and during a follow-up at 12 months postintervention.

Participant costs will be gathered through questionnaires during routine visits and the endline survey over the trial period. To assess the cost-effectiveness of the intervention, we will evaluate the incremental cost of providing eyeglasses to the intervention group. This includes the total cost of each pair of eyeglasses, incorporating potential replacement expenses, as well as the unit costs of screening, delivery and monitoring. We will then integrate costs and QALY in a within-trial cost-utility analysis.

### Handling of missing data

Every effort is made to minimise missing baseline and outcome data in the trial. An important consideration is that data unmeasured because of a participant’s death will not be considered as missing but rather as undefined. Missing data sensitivity analyses will be conducted to explore the sensitivity of the results to different assumptions about the missingness mechanism.

### Monitoring

Routine monitoring is carried out by LVPEI CTU on behalf of the sponsor, QUB. The research manager completes all required source documentation before the scheduled monitoring. The monitor educates relevant personnel on the protocol, reviews source documents, confirms that study documentation is complete, queries resolutions, reviews reporting of adverse events (AEs) and completes central monitoring activities, ensuring that data appear to be valid using range checks, no primary outcome data are missing and case report forms are submitted regularly. The registered trial protocol (ClinicalTrials.Gov, NCT05510687) describes the constitution and roles of the Trial Steering Committee and Data Monitoring and Ethics Committee in detail.

### Qualitative methods

Conducting a mixed-methods trial enhances understanding of the complex sociocultural and environmental contexts of the trial that may not be captured through quantitative methods alone.^33^ A formative, qualitative substudy is being conducted in the same setting as the trial to provide in-depth insights into cultural and contextual factors, barriers and facilitators that might influence the trial implementation and outcomes. Semistructured interviews are conducted with beneficiaries, implementers and policymakers associated with the social safety net programmes. Resulting data will be analysed using thematic analysis or qualitative content analysis to identify the key themes that may help refine the intervention and develop process evaluation measures. Results will be integrated with trial findings to provide a solid understanding of the intervention’s mechanisms and applicability in real-world settings, useful for future scale-up efforts.^34^

### Patient and public involvement

This study was conceived as mixed-method research, and we deliberately designed it to involve patients and other stakeholders at different points throughout the study. During the initial formative phase, we engaged members of the study population from our research areas to qualitatively understand the prevailing constraints and barriers to using DFS tools to receive social safety net allowances. We further scrutinised reasons for not using eyeglasses to correct presbyopia. We arranged workshops in the field to develop a ‘theory of change’ which guided the design of our study protocol. In addition to the social safety net beneficiaries, the workshops also included social safety net officers, field-level bureaucrats and local government leaders. The research team is working with experts on audio-visual content development to prepare accessible knowledge translation materials. We hope to reach out to a larger, non-technical audience once the trial results are peer-reviewed and published.

## Ethics and dissemination

Ethics approval was applied and received from QUB Medicine, Health and Life Sciences (MHLS) Research Ethics Committee (reference #MHLS22_69) and BRAC JPGSPH Institutional Review Board (reference #IRB-21 August’22-028). The trial is conducted in accordance with the principles of the Declaration of Helsinki and the International Conference on Harmonisation Good Clinical Practice Consolidated Guideline (E6), relevant regulations in Bangladesh and LVPEI CTU’s Standard Operating Procedures. Any episode of non-compliance will be documented. The study results will be released in open-access, peer-reviewed journals.

### Adverse reaction and safety reporting

The period for reporting AEs in the trial commences when informed consent is received from a participant and ends at the participant’s endline survey. All AEs are recorded and reported using study-specific forms in compliance with Bangladesh guidelines. All AEs are reported annually to the EC/IRB in the study progress report by the principal investigator.

### Participant confidentiality

Participant confidentiality will be ensured throughout the trial using a unique participant identification code (ID number) that will allow identification of all data reported for each participant. Data will be made publicly available only to the extent permitted by the applicable local laws and regulations. The mobile application that tracks financial transactions will not collect password or account information; thus, it does not present a risk of information being misappropriated which could lead to financial loss.

### Dissemination plan

Results from the primary and secondary outcomes will be reported for publication in open-access, peer-reviewed academic journals. The primary publication from this study will report the results of the study in accordance with the current ‘Uniform Requirements for Manuscripts Submitted to Biomedical Journals’ as established by the International Committee of Medical Journal Editors (www.ICMJE.org)

### Data sharing statement

The study complies with the good practice principles for sharing individual participant data from clinical trials,^35 36^ and data sharing will be undertaken in accordance with the required regulatory requirements. Only deidentified data will be shared with all the researchers for analysis. In the event of publications arising from such analyses, those responsible will need to provide the principal investigator with a copy of any intended manuscript for approval prior to submission.

All individual participant data (IPD) will be stored in anonymised format. Any IPD that includes personal identifiable information will be deleted when the data collection process is complete and before the initiation of data analysis.

## supplementary material

10.1136/bmjopen-2024-085083 online supplemental material 1
