# Supplementary material for: Transforming Households with Refraction and Innovative Financial Technology (THRIFT): study protocol for a randomised controlled trial of vision interventions and online banking among the elderly in Kurigram
Source: BMJ Open. 2024 Dec 23;14(12):e085083. doi: 10.1136/bmjopen-2024-085083 (PMC11667281; doi:10.1136/bmjopen-2024-085083)
Supplement: online supplemental material 1 [file bmjopen-14-12-s001.docx]

**Baseline data collection, Randomization, and Eyeglasses distribution**

“***T***ransforming ***H***ouseholds with ***R***efraction and ***I***nnovative ***F***inancial ***T***echnology (**THRIFT**) Study”, WP3

**PARTICIPANT INFORMATION SHEET FOR BASELINE SURVEY(WP3)**

**Protocol Title: T**ransforming **H**ouseholds with **R**efraction and **I**nnovative **F**inancial **T**echnology: A randomised controlled trial on the impact of free reading glasses to support use of smartphone mobile banking in Bangladesh among elderly recipients of government Old Age and widow Allowance payments.

**Application No**.: IRB-21 August'22-028

**Sponsor:** Queen’s University Belfast (QUB)

**Protocol number:** MHLS_22_69

**Principal Investigators**: Prof Atonu Rabbani, Prof Abu Shonchoy, and Prof Nathan Congdon

**Date**:

1. **What you should know about this study:**

- You are being asked to read this information leaflet because you may meet the requirements for taking part in this randomised trial that involves research.
- This consent form may contain words that are unfamiliar to you. Please ask our research staff to explain any words or information that you do not clearly understand.
- You are encouraged to ask questions and to discuss the study with the research team. If, after reading this form and having all your questions answered, you would like to participate, you will be asked to sign a consent form. The research staff would provide you with a copy of the signed form.
- The Study team will tell you in a timely manner about any significant new information which might make you want to stop being in the study. The study team will have you read and sign a new informed consent form to inform you of any new information.

1. **Why is this research being done?**

We are doing this study to learn if providing free reading glasses supported by training on mobile phone use and digital financial services can help old age allowance and widow allowance beneficiaries of Bangladesh to use the Digital Financial Services banking platform better.

1. **Why are you being approached for this study?**

You are being asked to participate in this study because you are an OAA/WA beneficiary with no mobile phone to use the mobile banking services on your own and have poor near vision that can be corrected with glasses.

1. **What will happen if you join this study?**

We must get your signature or thumb print on the consent form to do tests to learn if you are a good candidate for the study. If you are found to be a good candidate for the study, then you will be one among the 484 participants of the study. Your participation in the study will last up to 12 months. But if you have any other severe eye-related problems (such as cataract) that cannot be resolved with eyeglasses or if you cannot/do not provide consent, or if you do not pass the screening tests that test your ability to identify numbers, have the required skill to use the mobile phone and cognition test, then you will not be included in the study.

If you are eligible to take part in THRIFT, we’ll ask you to fill in a few more questionnaires, each of which vary in length and take between one and a half to 2 hours to complete. on lifestyle habits like what you eat, quality of life, your general health, how well you can move about and can concentrate and think, access to eye care and if you are able to take household decisions on your own. The questions will be easy to understand and answer. We will ask you to answer the questions honestly.  If you become tired or upset, you can tell the study staff; they will give you breaks as needed and continue when you are ready or you may also stop and return on another day when you feel comfortable.

Our research team will wear a mask and follow all covid rules during these activities.

After these tests are done, you will be put in either control group or case group at random like flipping a coin. If you are in the control group, you will receive a mobile phone and smartphone usage instruction at the beginning of the study and you will receive eyeglasses, smartphone usage training and mobile banking app training at the end of the trial.

If you are in the case group, you will receive mobile phone, eyeglasses, training on how to use your smartphone and mobile banking application at the beginning of the study.

You can choose the pair of glasses that suit your lifestyle and requirement. Our team will explain how to get used to with new glasses. For example, for the first few days you use your glasses, be careful when walking; it takes time to get used to seeing with them on. Most people become used to wearing their glasses within a week. Please remember that this pair of eyeglasses is good for near work, however not useful for the distant vision. You should report any problems, such as the glasses break or are uncomfortable to wear, to the project coordinator. We will give you another pair of glasses if yours break or are lost during the study.

We will also provide you with, free data to access mobile bank to use your money.

In addition, we will collect information related to your mobile banking usage from the mobile bank (bKash) during the research period. However, this information will only be used for research purpose and without permission from you and bKash, we will not disclose your personal identity and information with anyone outside the research group.

We have two research teams,

1.VisionSpring team that tests your eyes and provides the glasses

2. MOMODa Foundation team gives you free mobile phone and teaches you how to use the mobile phone to access your mobile bank.

The first visit by VS team to test your eyes and know your power may need 30 minutes approximately. They will visit you again after one month and after 5 months after giving you the glasses to check the condition of your glasses. Each of these visits may take 10 minutes.

Our research Team from MOMODa Foundation will visit you 4 times. After the first visit, the second visit will be after 3 months, 6 months and 9 months after providing you with the mobile phone. These visits are to help you with any problems in using the mobile phone. These visits may require 10 to 15 minutes each. The final visit by MOMODa team is at the end of the study when you will be asked to respond to some questions. These questions are similar to the first visit and this may take one to one and half hours.

If you become tired or upset, you can tell the study staff; they will give you breaks as needed and continue when you are ready or you may also stop and return on another day when you feel comfortable.

1. **What are the risks from being in the study?**

 The tests and the interviews will take about 2 hours. If you feel tired, you can rest and then continue.

1. There is a small risk that personal information you give us is seen by other people. We will make this unlikely by collecting only a small amount of private information and following BRAC JPG rules for taking care of such information.
2. Risks presented in our study, including the use of smartphones, and undergoing a routine vision test, are minimal and not more than one “encounters in everyday life”.
3. Wearing glasses will not harm you or your eyes. There may be some temporary discomfort, but this will not have any effect on your eyes.

The survey might take around one and half hours for you to complete. If you become tired or upset, you can tell the study staff; they will give you breaks as needed and continue when you are ready or you may also stop and return on another day when you feel comfortable.

1. The mobile application that tracks your financial transactions will not collect password or your account information thus, it does not present a risk of information being misused which could lead to any financial losses for you. However, you may be watchful for sluggish performance of the mobile phone (e.g., crashing of apps, freezing of the screen, unexpected restarts).
2. **Are there benefits to being in the study?**

- Even though there will be no direct benefits from your participation, your valuable information will help us understand the OAA program and WA program in Bangladesh. We hope that the results from our research will play an important role in improving your experience in receiving OAA and WA allowance through mobile financial services.
- While there is no direct benefit to you from this research, you may find this study helpful. No promise or guarantee of benefits is being made to encourage your participation.
- Everyone who is in the Case or Intervention group will get free glasses, free eye tests, free mobile phone, and training to use the mobile phone and mobile banking. All control group participants will also receive free eye test, free mobile phone and instruction on using smartphone. However, control group participants will get free glasses and training to use the mobile and mobile banking at the end of the study.
- You will receive free top up of your mobile phone data, each month during the study irrespective of the case-group assignment, on your usage of the mobile phone for the banking transactions.

1. **Do you have to join the study?**

No, Joining this study is not mandatory.

1. **Will it cost you anything to be in this study?**

No, there are no costs to you.

1. **Will you be paid if you join this study?**

- No, you will not be paid to participate in this research, however, the eye tests will be provided at no cost to you.
- If you are in the Case or Intervention group, you will get free mobile phone, free data, and training on using the mobile phone. If you are in the control group, you will also receive free eye test and mobile phone. However, control group participants will get and free glasses and training at the end of the study.

1. **Can you leave the study early?**

Yes, you may leave the study at any time. Just tell one of the study staff you want to stop. The permission you give us to use what we learn about you will continue until you cancel it. If you do that, you must leave the study and no new information will be gathered from you. What we learn about you before that date, however, can be used for the purpose of the study. However, your personal identification information will not be revealed at any stage. Study personnel who are permitted, will have access to your name and ID number until data collection is finished. After this, we will delete your contact information and other data that will identify you. You can request to access your study -related information if needed, only until this time.

1. **Why might we take you out of the study early?**

You may be taken out of the study if staying in the study could harm you; or you need a treatment that is not part of the study; or you do not follow study instructions; or the study is cancelled. There may also be other reasons we do not know at this time that would cause us to take you out of the study.

1. **What information about you will be kept private and what information may be given out?**

The research implementation partner BRAC JPGSPH will not share any information that can be used to identify you, and that any data that is analysed will be anonymised. We will follow the rules that the BRAC JPGSPH uses to protect information that identifies you. What we learn about you will be used only for research and training purposes by BRAC JPGSPH and its partners. Nothing that shows who you are will be given to anyone. The data that needs to be transferred to our partners at L.V.Prasad Eye Institute in India will be anonymized.

Information that does not identify you will be stored for 10 years following rules at the BRAC University in Bangladesh and national guidelines and as required by the funding body Wellcome Trust. This data will be accessible only to the Head of the Study and will be destroyed after 10 years following national guidelines.

1. **What information about you may be used or given out in this study?**

Things we learn about you during this study include your answers to the first questions and tests done in your home (to learn if you are eligible to join the study); and your answers to later questions and tests done during the study. This also includes what we learn about you during study visits, phone calls or surveys.

1. **Who may use and give out information about you?**

Everything we learn about you will be kept private. During the study, the research staff will take all precautions to protect any information that may identify you. Subject to your consent, the implementation partners will receive information to identify you so that they can invite you to receive the intervention.

1. **Who may see your health information?**

BRAC JPGSPH will keep your name, contact and personally identifiable information confidential and will not pass this information to any partners outside of Bangladesh.  BRAC JPGSPH will use this information as needed, to contact you about the research study, and make sure that relevant information about the study is recorded for your care, and to oversee the quality of the study.

Certain individuals from BRAC JPGSPH and regulatory organisations may look at your research records to check the accuracy of the research study. Our research partners outside of Bangladesh will only receive information without any identifying information.  The people who analyse the information will not be able to identify you and will not be able to find out your name, contact or personally identifiable information.

When you agree to take part in a research study, the information related to your research records may be provided to researchers running other research studies in this organisation and in other organisations.  MOMODa Foundation collects your social and personal information, VisionSpring collects information about your eye, JPG BRAC university has all the information that is collected as part of the study including the digital transactions. JPG BRAC removes all information that may personally identify you, before sharing the data with other organisations outside of Bangladesh including QUB, Florida International University, L.V. Prasad Eye Institute Clinical Trials Unit and Good Business Lab. These organisations may be universities and organisations involved in health care research in Bangladesh or abroad.  Your information will only be used by organisations and researchers to conduct research in accordance with Bangladesh’s policies on research related to digital financial transactions using smart phone application. This information will not identify you and will not be combined with other information in a way that could identify you.

1. **Why will this information be used and given out?**

What you tell us will be used during the study and to understand the results. Your information may be used in reports the government requires from us.

1. **How will information be used?**

The information will only be used for the purpose of research and cannot be used to contact you.

1. **What if you decide not to give your permission to use and give out your digital transaction based on the app?**

You can decide not to let us use or share your transactions done digitally through the app/smart phone. But in that case, you cannot join the study.

1. **How long does this privacy authorization last?**

The permission you give us to use what we learn about you will continue until you cancel it. If you do that, you must leave the study and no new information will be gathered about you. What we learn about you before that date, however, can be used.

1. **Is your information protected after it has been given to others?**

BRAC JPGSPH has agreements with other groups about how they use research related information. However, any data shared with others will be anonymized and will not personally identify you.

1. **What does a conflict of interest mean to you as a participant in this study?**

This is when a person or organisation would benefit from doing something that could influence their actions. The study staff and leaders of this project do not have any conflict of interest.

1. **What do you do if you have questions about the study?**

For any questions about the study, you may please contact, Prof Atonu Rabbani, Professor, BRAC James P Grant School of Public Health, on +880 1730-441787 or atonu.rabbani@bracu.ac.bd.

You may contact the IRB if you have questions about your rights as a participant or if you think you have not been treated fairly.

The Ethics Committee: BRAC JPG School of Public Health, BRAC University

Contact person: IRB administrator

Phone: XXXXX

Email: [irb-jpgsph@bracu.ac.bd](mailto:irb-jpgsph@bracu.ac.bd)

1. **What does your signature on the consent form mean?**

When you sign the Consent form, you have not given up any legal rights. Signing it means you understand the information on this form; you accept what the information tells you; and you agree to join the study.

**CONSENT FORM FOR BASELINE SURVEY Participant ID No. __________**

| **Title of Project:** | THRIFT Trial |
| --- | --- |
| **Chief Investigators:** | Dr. Nathan Congdon Study Number: MHLS22_69 |

**Put your initials or an ‘X’ in the box if you agree.**

| 1. | I confirm that I have read, or had read to me, and understand the information sheet dated 22 Nov 2023, version 7 for the above study. I have had the opportunity to ask questions and these have been answered fully. |  |
| --- | --- | --- |
| 2. | I understand that my participation is voluntary, and that I can leave the study at any time without giving any reason and without any consequences. |  |
| 3. | I understand the study is being conducted jointly by researchers from BRAC University in Bangladesh, and Queen’s University Belfast in the UK, and that my personal information will be held securely at BRAC University following data protection legislation. |  |
| 4. | I understand that data collected as part of this study may be looked at by authorized individuals from BRAC University or Queen’s University. I give permission for these individuals to have access to this information. |  |
| 5. | I understand that information I provide may be included in reports or publications, and in such a way that it will not be possible to identify me in such documents. |  |
| 7. | I understand that what is discussed during interviews or other data collection sessions is confidential, with the exception that if I disclose information that indicates that I am at risk of harming myself or others, or in danger of being harmed by someone else. In such cases the researcher is legally obliged to break confidentiality and pass on this information to the research supervisor. |  |
| 8. | I understand that information that can identify me will be retained until the collection of data is complete and I can access this information only until this time. I give researchers permission to store identifiable information until they have finished with data collection. |  |
| 9 | I give the researchers permission to store my information for 10 years so it may be used for future research or educational purposes in such a way that it will not be possible to identify me |  |
| 10 | Do you consent for your anonymous data to be used for future research or  Yes  ssssss  educational purposes? | No  No |
| 11 | Do you consent to being contacted in the future for follow-up and similar  Yes  ssssss  studies? |  |

If you agree with each of these statements, please answer the following question by putting an X on the box with your answer:

| Would you like to join the THRIFT study? | Yes | No |
| --- | --- | --- |
|  |  |  |

Participant’s Name (printed) Signature or Thumbprint Date

_______________________________ ________________________________

Enumerator’s’s Name (printed) Signature Date

_______________________________ _________________________________

**WE WILL GIVE YOU A COPY OF THIS SIGNED AND DATED CONSENT FORM.**
