# Supplementary material for: Transforming Households with Refraction and Innovative Financial Technology (THRIFT): study protocol for a randomised controlled trial of vision interventions and online banking among the elderly in Kurigram
Source: BMJ Open. 2024 Dec 23;14(12):e085083. doi: 10.1136/bmjopen-2024-085083 (PMC11667281; doi:10.1136/bmjopen-2024-085083)
Supplement: Supplementary file 2 [file bmjopen-2024-085083.reviewer_comments.pdf]

PEER REVIEW HISTORY

BMJ Open publishes all reviews undertaken for accepted manuscripts. Reviewers are asked to complete a checklist review form and are provided with free text boxes to elaborate on their assessment. These free text comments are reproduced below.

ARTICLE DETAILS

Title (Provisional)

Transforming Households with Refraction and Innovative Financial Technology (THRIFT): Study protocol for a randomised controlled trial of vision interventions and online banking among the elderly in Kurigram

Authors

Shitol , Sharmin Akter; Aftab , Ishrat Binte; Piyasena, Prabhath; Lohfeld, Lynne; Rayasam, Sridevi; Challa, Nagamani; Sangani, Payal; Sigwadhi, Lovemore Nyasha; Rahman, H. M. Masudur; Khanna, Rohit C; Chan, Ving Fai; Barua, Mrittika; Pant, Sonia; Adhvaryu, Achyuta; Nyshadham, Anant; Sarker, Malabika; Mettla, Asha Latha; Haque , Enam; MacKenzie, Greame; Alam , Sadiq; Gudwin, Ella; Clarke, Mike; Shonchoy, Abu; Rabbani, Atonu; Congdon, Nathan

---

VERSION 1 - REVIEW

**Reviewer**	**1**
**Name**	**Uche , Nkechi**
**Affiliation**	**University of Nigeria, OPHTHALMOLOGY**
**Date**	**11-Apr-2024**
**COI**	**NONE**

The dates of the study should be included

The qualitative aspect of the research should be expanded since its a mixed methods investigator-masked randomized control trial (RCT).

**Reviewer**	**2**
**Name**	**Onyia, Onyinye**
**Affiliation**	**Federal University Teaching Hospital, Owerri, Ophthalmology**
**Date**	**30-Apr-2024**
**COI**	**I have no competing interest to declare**

Thank you asking me to review this manuscript.

This is a thoroughly written protocol paper on an interesting subject area which will add significantly to body of knowledge on the synergist impact of technology and good eye health on productivity in LMI Economies.

In addition, the authors have sufficient expertise to conduct the study given their academic and professional background as well as previous unpublished work in a related field; the impact of phone ownership on account transactions among OAA beneficiaries in Bangladesh.

The proposed robust stepwise data analysis and presentation protocol appears to account for the deducible natural human non-compliance to the study protocol and behavioural social determinants of health such as participant crossing over, seeking assistance with transactions, non-use of provided corrections etc.

However, a few minor clarifications are highlighted below -

In table 1, row 10 - Role of study participant in household decision making, based on the description, the data type seems more of categorical than continuous. Kindly re-confirm before final draft.

Patient and Public Involvement - In this section, the authors should specifically outline how they involved or will involve the participants and public in the protocol development process or during the trial respectively.

Did the authors consider giving the control group placebo lenses? This may mask the participants since all participants will receive smartphone training but not mobile banking app training. Placebo lenses might also reduce the number of participant in the control group who may source near correction elsewhere thereby reducing the number that may deviate, although data analysis will be by intention to treat.

VERSION 1 - AUTHOR RESPONSE

Reviewer: 1
Dr. Nkechi Uche , University of Nigeria

(i) The dates of the study should be included.

Response: The start date for the study is 10 Dec 2023 and anticipated end date is June 2025. These dates are provided in the revised protocol article as advised, under section “Trial Status”.
(ii) The qualitative aspect of the research should be expanded since its a mixed methods investigator-masked randomized control trial (RCT).
Response: Thank you for the feedback. The qualitative aspects of THRIFT have been expanded in the "Materials and Methods" section. Please see qualitative methods on page 14.

Reviewer: 2

Dr. Onyinye Onyia, Federal University Teaching Hospital, Owerri

(i) In table 1, row 10 - Role of study participant in household decision making, based on the description, the data type seems more of categorical than continuous. Kindly re-confirm before final draft.
Response: Thank you for your suggestion. We have updated the data type for row 10, "Role of study participant in household decision making," to categorical. Additionally, the table has been revised to ensure each outcome is classified as either continuous or categorical to avoid inconsistencies. Please see page 10-12.

(i) Patient and Public Involvement - In this section, the authors should specifically outline how they involved or will involve the participants and public in the protocol development process or during the trial respectively.
Response: Thank you for the suggestion. As mentioned in the editorial comments, details on patient and public involvement have been added to the "Methods" section. Please see page 14-15.

(ii) Did the authors consider giving the control group placebo lenses? This may mask the participants since all participants will receive smartphone training but not mobile banking app training. Placebo lenses might also reduce the number of participants in the control group who may source near correction elsewhere thereby reducing the number that may deviate, although data analysis will be by intention to treat.
Response: Thanks for this suggestion, which makes good scientific sense! However, providing placebo eyeglasses without power to the elderly participants was not ethically acceptable in the view of the investigators and local stakeholders. This would lead study participants to erroneously conclude that glasses have no benefit, and risk undermining demand for vision services in the area.

This rationale has been added to the "Study Intervention" section of the article. Please see page 8-9.
